# Agile response to the shortage of personal protective equipment during the COVID-19 crisis

**DOI:** 10.6061/clinics/2020/e2281

**Published:** 2020-11-02

**Authors:** Marcos Moraes, Renata Pivi de Almeida, José Eduardo Lopes da Silva, Marisa Riscalla Madi, Dirceu Carrara, Marcia Hitomi Takeiti, Tânia Mara Varejão Strabelli, Vilson Cobello Junior

**Affiliations:** I Universidade de Sao Paulo Instituto do Coracao (InCor-HC-FMUSP); II Universidade de Sao Paulo Hospital das Clinicas (HC-FMUSP)

The severe acute respiratory syndrome coronavirus (SARS-CoV-2) caused one of the biggest health crises in history. Due to the overload on the health systems, the potential for transmission in the hospital setting was almost twice as high as in the community (1). The uncertainty regarding the behavior and impact of the new disease determined the increased demand for personal protective equipment (PPE) at the *Instituto do Coração do Hospital das Clínicas da Faculdade de Medicina da Universidade de São Paulo* (InCor-HC-FMUSP), a tertiary hospital with 3700 employees and 445 beds, of which 168 are in the intensive care unit.

Although there is no evidence in the literature that the use of N95 respirators, when compared to the use of surgical masks, provides greater protection to health workers when performing non-aerosol generating procedures (2), the demand for N95 respirators increased exponentially with the outbreak of COVID-19 in São Paulo in March 2020. This also raised the demand for surgical masks, hand sanitizers, liquid alcohol, and goggles. Additionally, there was a shortage of these items throughout the supply chain, leading to abusive prices and shortage in the national and international markets. This imposed the need to implement rigorous mechanisms for rationalizing the use in InCor-HC-FMUSP.

## Tackling the shortage: educating healthcare workers and controlling the dispensation

Similar to what happened in other countries, in the first weeks of the pandemic, there was a great wastage of these items, either due to misuse or fear of scarcity that led to individual storage by some employees, leading to more scarcity for others. To address these problems, a multi-professional planning team was formed and met daily for a week, with actions concentrated on two pillars: employee education and dispensation control.

For employee education, good practices for the use of PPE were disseminated, as contained in the manual for its use at the HC-FMUSP through the institutional media, with clarifications on the adopted rules and guidelines. Additionally, the Hospital Infection Control Unit promoted training in several units of the hospital, explaining how, when, and where each type of PPE should be used alongside training the teams in the techniques of dressing and undressing, thus mitigating the risk of contamination among the employees (3).

To rationalize the dispensation and, thus, guaranteeing the supply of adequate PPE to the health workers who would really benefit from its use, three PPE distribution centers were installed: two with daily operations of 14 h located near the intensive care units and the wards, and one with a 24-h operation, 7 days a week, next to the emergency department. Volunteers from different sectors of the hospital were recruited and trained. With the help of the coordination team, their work schedule was structured organically to operate these distribution centers.

The units started their operations with a few resources: a shared physical space with other sectors of the Institute and a shared spreadsheet from Google^®^ as a means of control. Based on the distribution and apportionment criteria defined by the planning team, volunteers manually entered the withdrawal date of each item in the list of employees. This model allowed the immediate start of distribution control; however, there was a need for further automation in order to optimize the process. To this end, while the units were already carrying out the control manually, a system for checking and automatic registration of item withdrawals through a badge reader was developed by the Corporation Center for Information Technology (NETi) and implemented within three weeks. With this improvement, in addition to the employee's photo appearing on the screen, the date and time of the withdrawal were recorded alongside the system’s warning about the day and time of the next potential withdrawal, thus favoring control and avoiding over-dispensation.

## Agile methodologies as an alternative (and necessity) to traditional project management methods during the crisis

Traditional project management methods are characterized by being highly predictive. They start with an accurate requirement specification that once defined, cannot be changed during the execution of the project. Additionally, each designed step needs to be completed before moving on to the next. The main problem is specifying all the possible requirements with a high level of detail before the initiative starts for real-life projects. Since they are rarely sequential and carry a certain amount of requirement uncertainties that demand changes during the journey *i.e.* by following this model solely and systematically, there may be no way to improve the initial imperfections in one of the later phases of the project. It is exactly in this field that Agile Methodologies gain prominence when they allow a management mechanism that ensures controlled changes without affecting the product and its progress (4).

Agile models were designed for adaptability. A product in an agile project is developed in a series of short-term rounds (iterations), and each iteration demands requirement gathering, requirement elicitation, planning, design, coding, and testing. Therefore, this framework guarantees no wasted time and effort in the planning phases of the project because the shorter the deadline, the easier it becomes to predict the number of requisites and increments that will be necessary for the next version. Meanwhile, a valuable working part of the product is delivered to the customer at the end of each iteration (4). This adaptability of the agile philosophy is best suited to a scenario full of uncertainties, such as the actual pandemic period.

During the operation of the distribution centers, the opening hours and guidelines established on the recommendations for the use of each PPE were frequently reassessed considering the available stock of the items, suppliers' response, and amount of masks and hand sanitizers received as donation, to provide greater flexibility in the delivery. In addition to the constant re-evaluations of protocols for the use of each PPE, the user flexibility and quantity of items dispensed could also be requested by the leader of each area, if formally justified by e-mail and after evaluation by the planning team.

From this experience, two conclusions are evident: the first is the successful implementation of the centralization of PPE distribution using an IT solution as a tool to control and track consumption, mainly as a barrier to evasion and inappropriate use of materials, and also as a way to educate employees about the correct use of available resources. The second conclusion is the probable inability to implement a project like this, in a chaotic context like the COVID-19 crisis, using only traditional project management methods, which take longer and require excessive meetings between the involved parties. As described in the literature, in periods of high unpredictability, it is not appropriate to attempt to decide all the details of a new product with such a high impact on the institution without working with feedback and continuous improvement cycles.
